# MULTI OMICS LANDSCAPE OF INSULIN SENSITIVITY IN HEALTHY OLDER PEOPLE FROM THE LONG LIFE FAMILY STUDY COHORT

**DOI:** 10.1093/geroni/igad104.3480

**Published:** 2023-12-21

**Authors:** Vaha Akbary Moghaddam, Shu Liao, Bharat Thyagarajan, Kaare Christensen, Gary Patti, Michael Brent, Michael Province, Ping An

**Affiliations:** Washington University in St. Louis, St. Louis, Missouri, United States; Washington University in St. Louis, St. Louis, Missouri, United States; University of Minnesota, Minneapolis, Minnesota, United States; University of Southern Denmark, Odense, Syddanmark, Denmark; Washington University in St. Louis, St. Louis, Missouri, United States; Washington University in St. Louis, St. Louis, Missouri, United States; Washington University School of Medicine, St. Louis, Missouri, United States; Washington University in St. Louis, St. Louis, Missouri, United States

## Abstract

Insulin sensitivity (HOMA-S), which measures the body’s response to insulin, is a crucial indicator of glucose homeostasis, particularly in healthy people. The present study utilizes MultiOmics profiling and integration to explore molecular processes crucial for maintaining healthy HOMA-S. To this end, 3783 non-diabetic individuals from 567 pedigrees in Long Life Family Study Cohort were selected (Average age = 70±16). Upon adjustment of HOMA-S for relevant covariates, linear mixed models were employed to analyze the genome sequence, blood transcriptome, and metabolome associations with HOMA-IS. Moreover, using STAAR and Pascal packages, rare and common variants were collapsed to genomic co-ordinates. The results highlight 10 genes, 2 soluble metabolites, and 2 lipids (phosphatidylcholine 35:1 and 40:6), significantly associated with HOMA-S. Integrative module enrichment analysis with PascalX package, identified multiple significant networks for HOMA-S. Notably, immune response in asthma is one of the processes with most genes (57%) displaying a positive correlation with HOMA-S (module p-val = 1.89 × 10E-6). The network contains 2 novel genes from transcriptome analysis, FCER1A and MS4A2 (p-vals = 1.32 × 10E-8 and 1.42 × 10E-6). Interestingly, one of the key metabolites, N-acetylglycine, also has a strong association with MS4A2 (p-val = 0.001034). Furthermore, SNP colocalization for gene expression, metabolome peak intensities, and HOMA-S, revealed multiple significant signals simultaneously associated with PC 35:1 and two members of this network, HLA-DQA1 and HLA-DQB1. The results of the study underscore the intricate interplay of genetic and metabolic factors for healthy HOMA-S with aging and their potential protective role for glycemic implications.

